# Normalizing to GADPH jeopardises correct quantification of gene expression in ovarian tumours – IPO8 and RPL4 are reliable reference genes

**DOI:** 10.1186/1757-2215-6-60

**Published:** 2013-08-30

**Authors:** Zuzana Kolkova, Arsen Arakelyan, Bertil Casslén, Stefan Hansson, Eva Kriegova

**Affiliations:** 1 Department of Obstetrics & Gynaecology, Lund University, Skåne University Hospital Lund, Lund, SE 221 85, Sweden; 2 Institute of Molecular Biology, NAS RA 7 Hasratyan St, Yerevan 0014, Armenia; 3 Department of Immunology, Faculty of Medicine and Dentistry, Palacky University, Olomouc, Czech Republic

## Abstract

**Background:**

To ensure a correct interpretation of results obtained with quantitative real-time reverse transcription-polymerase chain reaction (RT-qPCR), it is critical to normalize to a reference gene with stable mRNA expression in the tissue of interest. GADPH is widely used as a reference gene in ovarian tumour studies, although lacking tissue-specific stability. The aim of this study was to identify alternative suitable reference genes for RT-qPCR studies on benign, borderline, and malignant ovarian tumours.

**Methods:**

We assayed mRNA levels for 13 potential reference genes – ABL1, ACTB, CDKN1A, GADPH, GUSB, HPRT1, HSP90AB, IPO8, PPIA, RPL30, RPL4, RPLPO, and TBP –with RT-qPCR in 42 primary ovarian tumours, using commercially pre-designed RT-qPCR probes. Expression stability was subsequently analysed with four different statistical programs (GeNorm, NormFinder, BestKeeper, and the Equivalence test).

**Results:**

Expression of IPO8, RPL4, TBP, RPLPO, and ACTB had the least variation in expression across the tumour samples according to GeNorm, NormFinder, and BestKeeper. The Equivalence test found variation in expression within a 3-fold expression change between tumour groups for: IPO8, RPL40, RPL30, GUSB, TBP, RPLPO, ACTB, ABL1, and CDKN1A. However, only IPO8 satisfied at a 2-fold change as a cut-off. Overall, IPO8 and RPL4 had the highest, whereas GADPH and HPRT1 the lowest expression stability. Employment of suitable reference genes (IPO8, RPL4) in comparison with unsuitable ones (GADPH, HPRT1), demonstrated divergent influence on the mRNA expression pattern of our target genes − GPER and uPAR.

**Conclusions:**

We found IPO8 and RPL4 to be suitable reference genes for normalization of target gene expression in benign, borderline, and malignant ovarian tumours. Moreover, IPO8 can be recommended as a single reference gene. Neither GADPH nor HPRT1 should be used as reference genes in studies on ovarian tumour tissue.

## Background

Most cases of ovarian cancer are diagnosed at an advanced stage, with poor prognosis for the patients. Early stages of ovarian cancer are, on the other hand, more accessible to treatment and have much better prognosis. There is an ongoing search for biomarkers with capacity to detect in particular early stages of the disease in screening programs, since this would be the single most important step towards improving the prognosis. A selective biomarker might, furthermore, be helpful in the preoperative assessment of ovarian lesions in order to employ optimal surgery.

Analysis of gene expression by quantitative real-time reverse transcription-polymerase chain reaction (RT-qPCR), a sensitive technique with broad dynamic range, is a frequent approach for the biomarker discovery in tumour tissue. However, in order to obtain reliable results by RT-qPCR in heterogeneous clinical samples, the expression of a target gene needs to be normalized to a stably expressed reference gene (RG) to minimize the influence of variations in, e.g. extraction yield, reverse-transcription yield, and amplification efficiency [1]. Stability of such reference genes has to be validated in benign and malignant tissues from the specific organ studied. Use of an unstable reference gene will inevitably produce erroneous results. Needless to say, this requirement applies also for ovarian tumours with different differentiation grades and histological types.

The traditionally used house-keeping gene, glyceraldehyde-3-phosphate dehydrogenase (GADPH), was reported to display many diverse activities unrelated to its glycolytic function (e.g. apoptosis and DNA replication) [2], and to be up-regulated in prostate cancer already in the 1990s [3]. The most commonly used RT-qPCR reference genes used for ovarian studies has been GADPH (~40%), β-actin (ACTB) (~20%), ribosomal RNA (18S and 28S rRNA) (~10%) and hypoxanthine phosphoribosyl transferase 1 (HPRT1) (<3%) [4]. More recent study has advised against the use of GADPH and ACTB as RG’s, due to their numerous pseudogenes present in the human genome [5].

Up to now, only two studies have focused on finding a reliable RG in normal ovarian tissue, and benign and malignant serous ovarian tumours. The obtained results, however, differ; Li *et* al. recommended combination of GUSB, PPIA, and TBP [4], whereas Fu *et* al. concluded that combination of RPL4, RPLPO, and HSP90AB1 (HSPCB) are more suitable [6]. Both studies were performed on Chinese populations, did not include borderline tumours, and used SYBR Green RT-qPCR technique.

The present study was performed on a Scandinavian population, included borderline tumours, used predesigned commercial RT-qPCR probes, and applied four different statistical software programs. In addition to the above mentioned traditionally used and earlier recommended RGs for ovarian tissue, we also selected four genes from a commercially printed array (ABL1, CDKN1A, IPO8, and RPL30). Thus, altogether 13 genes we included in the study. Finally, two target genes were chosen to demonstrate the divergent results, which may be obtained by normalizing their mRNAs to suitable vs. unsuitable RGs: G protein-coupled estrogen receptor (GPER), which has no differences in expression between benign and malignant ovarian tumours and urokinase plasminogen activator receptor (uPAR), which is up-regulated in malignant tumours.

## Methods

### Ovarian tumour tissue

Tissue samples (n = 42) were obtained from primary ovarian tumours during surgery at the Department of Obstetrics and Gynaecology, Lund University Hospital, during 2001–2007. None of the patients had received chemotherapy prior to the operation. The samples were cut in 5 × 5 × 5 mm cubes, quick frozen on dry ice, and stored at −80°C until used. In addition to the routine histo-pathological examination, each specimen was re-evaluated by a second pathologist. Histological differentiation was classified as benign (n = 9), borderline (n = 11), and malignant (n = 22); the histological types were serous (n = 21), mucinous (n = 13), and endometrioid (n = 8) (Table 1). The mean age of included patients was 59 years (range 22–80) in the benign group, 55 years (35–86) in the borderline group, and 62 years (43–85) in the malignant group. The Ethical Review Board at Lund University Hospital approved the study design and informed consent was obtained from each patient.

**Table 1:** Distribution of the primary ovarian tumours according to histopathology

	***Serous***	***Mucinous***	***Endometrioid***	***Total***
**Benign**	4	5		9
**Borderline**	6	5		11
**Grade 1**	6	2		8
**Grade 2**		1	3	4
**Grade 3**	5		5	10
**Total**	21	13	8	42

### Extraction of total RNA

Total RNA was extracted from about 125 mg frozen ovarian tumour tissue. The tissue was homogenized in Trizol 50 mg/mL (Invitrogen, Carlsbad, CA) using rotating-knives (Polytron). All RNA samples were checked for concentration and purity by NanoDrop Spectrophotometer ND-1000 (Saveen Werner, Limhamn, Sweden) having A_260/280_ and A_260/230_ ~ 2. RNA quality and integrity was verified by Agilent 2100 BioAnalyzer (Agilent Technologies, Palo Alto, CA), i.e. all samples had RNA Integrity Number > 7.7.

### cDNA synthesis

GeneAmp® RNA PCR kit (Applied Biosystems, Foster City, CA) was used for reverse transcription of total RNA (0.2 μg) to cDNA. The final concentration of cDNA was 1 μg/μL (+/− 7%) and A_260/280_ ratio ~1.8 as assessed by NanoDrop. The cDNA samples were stored at −20°C until further use.

### Quantitative RT-qPCR amplification

RT-qPCR was performed using a StepOnePlus™ cycler (Applied Biosystems) under standard thermal cycling conditions (activation of contamination preventing enzyme at 50°C for 2 min, enzyme activation at 95°C for 10 min, 40 cycles of denaturation at 95°C for 15 s, and annealing at 60°C for 1 min). PCR reactions were run in duplicates and negative controls were included in each amplification set. For each gene analysed, pre-manufactured real-time qPCR assays were used (Applied Biosystems or Integrated DNA technologies, Inc., Coralville, IA, USA) (Table 2), with probes spanning exon junctions and not detecting genomic DNA. Using one malignant tumour sample and a universal human reference RNA (Stratagene, La Jolla, CA, USA), quantification experiments were performed using two standard curves from 10-fold serial dilutions of the cDNA (80–0.08 ng).

**Table 2:** **Reference genes**, **target genes and assays used**

***Gene symbol***	***Gene name*(*synonyms*)**	***Function***	***NCBI Gene reference***	***Assay ID***
**ABL1**	C-abl oncogene 1, non-receptor tyrosine kinase	Cell differentiation, division, adhesion and stress response.	NM_005157.3, NM_007313.2	Hs00245445_m1
**ACTB**	Actin, beta	Cell motility, structure, integrity	NM_001101.3	Hs99999903_m1
**CDKN1A**	Cyclin-dependent kinase inhibitor 1A (p21, Cip1)	Regulation of cell cycle progression at G1.	NM_004064.3	Hs00355782_m1
**GADPH**	Glyceraldehyde-3-phosphate dehydrogenase	Catalysation of an important energy-yielding step in carbohydrate metabolism.	NM_002046.3	Hs99999905_m1
**GUSB**	Glucuronidase, beta	Degradation of glycosaminoglycans	NM_000181.2	Hs99999908_m1
**HPRT1**	Hypoxanthine phosphoribosyl transferase 1	Generation of purine nucleotides through the purine salvage pathway.	NM_000194.2	Hs99999909_m1
**HSP90AB1**	Heat shock protein 90	Protein folding, response to stress.	NM_007355	Hs.PT.49a.20846338
**IPO8**	Importin 8	Nuclear transport.	NM_001190995.1 NM_006390.3	Hs00183533_m1
**PPIA**	Peptidylprolyl isomerase A (cyclophilin A)	Protein folding, ligand for Cyclosporin A.	NM_021130.3	Hs99999904_m1 Hs.PT.39a.22214851
**RPL30**	Ribosomal protein L30	Component of 60S subunit. Catalysation of protein synthesis.	NM_000989.2	Hs00265497_m1
**RPL4**	Ribosomal protein L4	Component of 60S subunit.	NM_000968	Hs.PT.49a.20266660
**RPLPO**	Ribosomal protein, large, PO	Component of 60S subunit.	NM_053275.3, NM_001002.3	Hs99999902_m1
**TBP**	TATA box binding protein	Initiation of transcription of RNA polymerases.	M34960.1 M55654.1	Hs99999910_m1
**GPER**	G protein-coupled estrogen receptor	Rapid estrogen signalling.	NM_001505.2	Hs00173506_m1
**uPAR**	Urokinase plasminogen activator receptor	Cell invasion, migration, signalling via ERK1/2.	NM_001005376.2 NM_001005377.2 NM_002659.3	Hs00182181_m1

### Identification of new potential reference genes

In order to identify new candidate reference genes in ovarian tumour tissue, we employed a commercial array (TaqMan® Express Endogenous Control Plate, cat no 4396840, Applied Biosystems) consisting of 32 potential RGs (18S, GADPH, HPRT1, GUSB, ACTB, B2M, HMBS, IPO8, PGK1, RPLPO, TBP, TFRC, UBC, YWHAZ, PPIA, POLR1A, CASC3, CDKN1A, CDKN1B, GADD45A, PUM1, PSMC4, EIF2B1, PES1, ABL1, ELF1, MT-AT6, MRPL19, POP4, RPL37A, RPL30, RPS17).

We analysed one benign and one malignant sample of ovarian tumour, which were selected based on the greatest difference in expression of traditionally used RGs (ACTB, GADPH, and HPRT1), as measured by RT-qPCR. The difference between the threshold cycles (ΔC_t_) of the two samples was then calculated for each of the 32 genes in the array. Four genes with the lowest ΔC_t_ were selected for inclusion in our main study.

### Statistical analysis

Descriptive statistics, F-test for C_t_ variance equality and Kolmogorov-Smirnov test for normality of log-transformed relative expression values were calculated by software SPSS 19.0 (SPSS Inc, Chicago, IL). The Equivalence test [7-9] and statistical applets BestKeeper [10], geNorm [11], and NormFinder [12] were used for analysis of genes expression stability. GeNorm calculates a gene-stability measure, M-value, as the average pair-wise variation of a particular gene to all other candidate reference genes [11]. On the other hand, the stability value calculated with NormFinder combines estimated both intra-group and inter-group variations [12]. Genes with the lowest M-values have the most stable expression (least variability). Relative expression values for target genes were analysed by Kruskal-Wallis and Mann–Whitney tests, and the log-transformed values by one-way ANOVA. P < 0.05 was considered significant.

## Results

### Selection of best RGs from the commercial gene array

In order to select optimal candidate RGs for this study on ovarian tumours, ΔC_t_ between one benign and one malignant ovarian tumour sample with the greatest difference in expression of the traditionally used RGs (ACTB, GADPH, and HPRT1), was measured by RT-qPCR and calculated for all 32 genes included in the arrays. The lowest ΔC_t_, i.e. the least variation, was found for CDKN1A (ΔC_t:_ 0.47), ABL1 (0.76), RPL30 (0.83), RPS17 (1.09), MT-ATP6 (1.42), and IPO8 (1.71), whereas POP4 (6.11), GADPH (5.04), HPRT1 (4.91), POLR2A (4.41), CASC3 (3.48) had the highest ΔC_t_. The most abundant genes were 18S (mean C_t_ ± SD: 12.11 ± 1.85) and MT-ATP6 (21.64 ± 1.00), the genes with lowest expression were YWHAZ (31.42 ± 2.14) and TBP (31.37 ± 2.06). CDKN1A, ABL1, RPL30 and IPO8 were chosen to be included in our panel of potential reference genes.

### Expression of selected candidate reference and target genes in primary ovarian tumours

**Figure 1:**
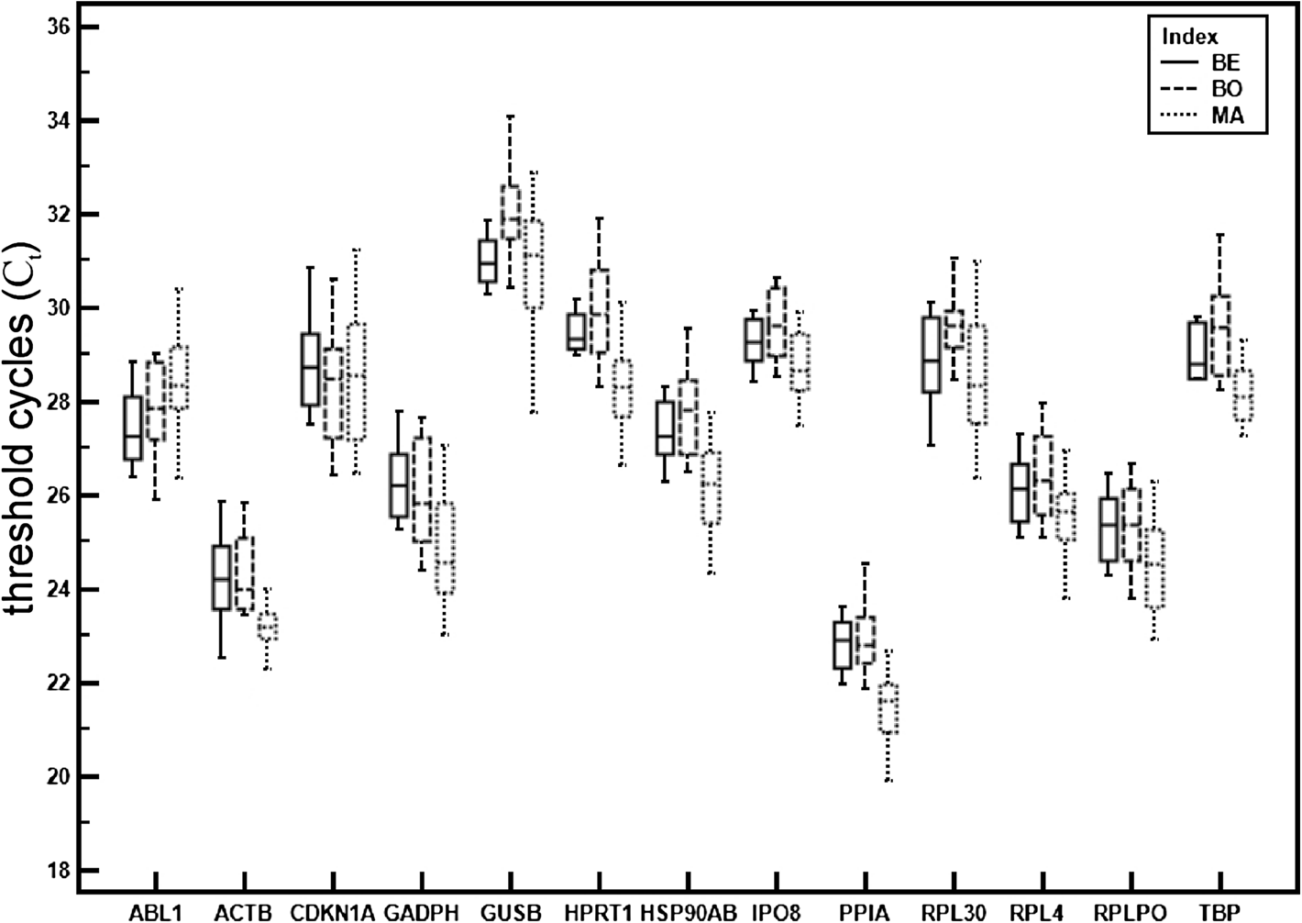
**Expression levels of 13 candidate reference genes in benign (BE), borderline (BO), and malignant (MA)primary ovarian tumours.** Values are given as the cycle threshold (C_t_) and are inversely proportional to the amount of template. Expression levels of the genes studied are shown as whiskers box plots.

We analysed altogether 13 candidate reference genes (ABL1, ACTB, CDKN1A, GADPH, GUSB, HPRT1, HSP90AB, IPO8, PPIA, RPL30, RPL4, RPLPO, and TBP) and two target genes (GPER and uPAR) by RT-qPCR. Expression levels and variability of C_t_ values are shown for the RGs (Table 3). Of all genes, PPIA had the highest (mean C_t_ ± SD: 22.12 ± 0.82) and GUSB the lowest (31.20 ± 0.99) level of mRNA (Figure 1). The amplification efficiencies of the TaqMan-based RT-qPCR assays were in the range 85–99% for all RGs, except ABL1 and HPRT1, which had 82% efficiency. The linear regression coefficient (r^2^) of the standard curves for all genes ranged between 0.998 and 1.

**Table 3:** Descriptive and correlation analysis of the candidate RGs obtained by BestKeeper

	**ABL1**	**ACTB**	**CDKN1A**	**GADPH**	**GUSB**	**HPRT1**	**HSP90**	**IPO8**	**PPIA**	**RPL30**	**RPL4**	**RPLPO**	**TBP**
**n**	41	42	42	41	42	41	42	42	42	42	42	42	42
**gM[C**_**t**_**]**	28.05	23.73	28.54	25.39	31.20	29.02	26.81	29.10	22.12	28.78	25.88	24.86	28.70
**aM[C**_**t**_**]**	28.07	23.75	28.57	25.42	31.23	29.04	26.84	29.11	22.15	28.81	25.90	24.88	28.71
**min[C**_**t**_**]**	25.90	21.80	26.43	23.02	27.75	26.63	24.30	27.48	19.91	26.34	23.79	22.91	27.28
**max[C**_**t**_**]**	30.39	25.87	31.23	27.80	34.06	31.91	29.55	30.64	24.53	31.06	27.98	26.66	31.55
**SD[± C**_**t**_**]**	0.87	0.73	1.05	1.05	0.99	0.91	0.86	0.65	0.82	1.09	0.77	0.81	0.75
**CV[% C**_**t**_**]**	3.10	3,07	3.69	4.11	3.17	3.13	3.19	2.22	3.71	3.78	2.98	3.27	2.62
**min[x-fold]**	−3.62	−3.36	−4.00	−4.33	−10.12	−4.17	−5.66	−2.76	−4.17	−4.53	−3.79	−3.37	−2.62
**max [x-fold]**	4.04	3.85	5.85	4.41	6.78	5.64	6.62	2.61	4.73	4.11	3.82	3.06	6.15
**SD [± x-fold]**	1.68	1.55	1.88	1.87	1.81	1.72	1.67	1.47	1.63	1.96	1.61	1.66	1.59

Geometric mean of C_t_ (**gM** [**C**_**t**_]), arithmetic mean (aM [**C**_**t**_]), minimum and maximum values of C_t_ (**min** [**C**_**t**_], **max** [**C**_**t**_]), standard deviation of C_t_ (**SD** [± **C**_**t**_]), coefficient of variance expressed as a percentage on the C_t_ level (**CV** [% **C**_**t**_]), extreme values of expression levels expressed as an absolute x-fold over- or under- regulation coefficient (**min** [**x**-**fold**], **max** [**x**-**fold**]), and standard deviation of the absolute regulation coefficients (**SD** [± **x**-**fold**]).

### Gene expression stability calculated by GeNorm

Expression stability of the 13 candidate RGs was first assessed by GeNorm in the whole set of tumour samples. The expression stability value (M-value) was calculated based on the average pair-wise variation between all genes tested (Table 4). The genes with the lowest M-value have the most stable expression and were ranked as follows: the most stable-IPO8 > RPL4 > TBP > RPLPO > ACTB > PPIA > HSP90 > HPRT1 > GADPH > ABL1 > CDKN1A > GUSB > RPL30.

**Table 4:** Ranking of 13 candidate RGs according to their expression stability by GeNorm and NormFinder

**GeNorm**		**NormFinder**	
**Gene**	**M-value**	**Gene**	**M-value**
IPO8	0.55	TBP	0.225
RPL4	0.55	RPLPO	0.251
TBP	0.58	IPO8	0.253
RPLO	0.60	ACTB	0.264
ACTB	0.62	RPL4	0.272
PPIA	0.65	PPIA	0.339
HSP90	0.67	HSP90	0.357
HPRT1	0.72	GADPH	0.373
GADPH	0.77	HPRT1	0.396
ABL1	0.86	CDKN1A	0.433
CDKN1A	0.93	RPL30	0.441
GUSB	1.00	GUSB	0.444
RPL30	1.10	ABL1	0.515

### Gene expression stability calculated by NormFinder

M-values were calculated for individual RGs using NormFinder that assessed the expression stability by combining estimated inter- and intra-group variation (Table 4). The genes were ranked according to expression stability as follows: the most stable-TBP > RPLPO > IPO8 > ACTB > RPL4 > PPIA > HSP90 > GADPH > HPRT1 > CDKN1A > RPL30 > GUSB > ABL1. The five best-ranked genes — TBP, RPLPO, IPO8, ACTB, and RPL4 — turned out to be the same five most stable genes found by GeNorm.

Moreover, NormFinder allowed stability analysis between subgroups: 1) benign, 2) borderline, 3) malignant, 4) serous benign and borderline tumours 5) mucinous, benign and borderline tumours, 6) serous malignant tumours, and 7) endometrioid malignant tumours (Table 5). Combining the two most stable genes further improved the M-value in group-wise comparison. In all obtained combinations, IPO8 followed by RPL4 came out as the most stable genes.

**Table 5:** **NormFinder ranking of 13 candidate RGs and combinations of the two best in group**-**wise comparison**

**Gene name**	**BE × BO × MA**	**BE+BO × MA**	**BE × BO+MA**	**BE × MA**	**Ser × Muc(BE+BO)**	**Ser × End (MA)**
ALB1	13	13	13	13	9	9
ACTB	2	4	6	5	7	4
CDKN1A	12	12	8	8	12	11
GADPH	8	9	11	11	10	10
GUSB	7	7	12	12	11	12
HPRT1	10	8	7	7	8	5
HSP90	6	11	5	6	2	8
IPO8	5	3	2	1	1	2
PPIA	9	10	9	9	4	3
RPL30	11	6	10	10	13	13
RPL4	1	1	3	2	3	7
RPLPO	4	2	4	3	6	6
TBP	3	5	1	4	5	1
Best combination	RPL4/ACTB	RPL4/RPLPO	IPO8/TBP	IPO8/RPL4	IPO8/HSP90	IPO8/TBP
M-value	0.104	0.088	0.060	0.079	0.060	0.073

### Analysis of expression stability by BestKeeper and equivalence test

In the next step, candidate RGs were evaluated by BestKeeper and the Equivalence test for variations in expression in the whole data set and between tumours groups as described above. IPO8 had the lowest standard deviation (SD) of the C_t_ value across the groups (mean C_t_ ± SD: 29.10 ± 0.65). The best-ranked genes by GeNorm and NormFinder — IPO8, ACTB, TBP, RPL4, and RPLPO — fulfilled the BestKeeper criteria for stability variation of the C_t_ value with SD < 1 (Table 3). GADPH had SD > 1 and hence did not meet the stability criteria.

**Figure 2:**
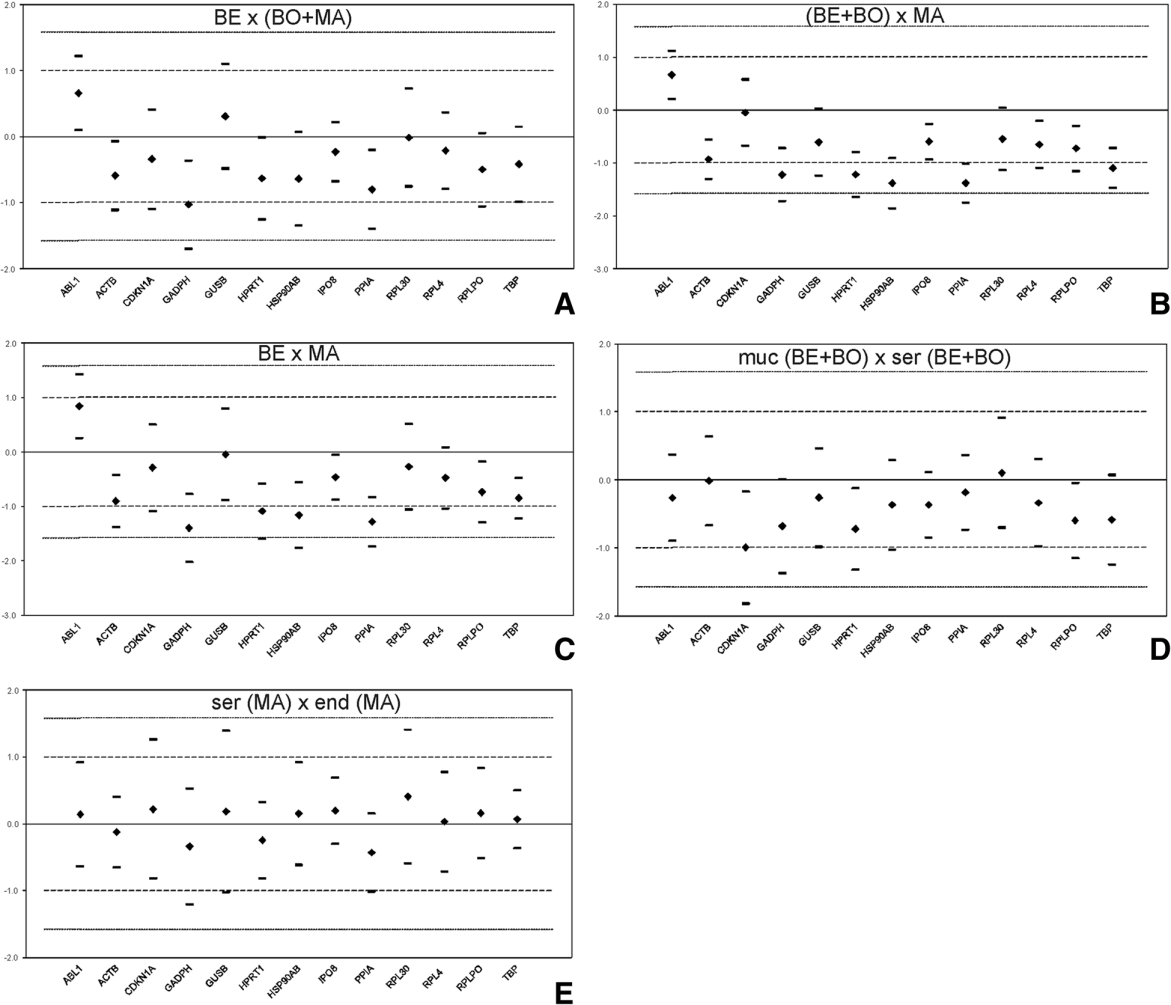
**Variation in expression of 13 candidate reference genes analysed by Equivalence test between tumour groups.** Differences of the means (♦) and matching symmetrical confidence intervals (-) are shown for the log2-transformed relative gene expression. Y-axis represents the fold change in expression among subgroups. The deviation area [-l; l] for a fold change ≤2 lies within the dashed lines; the deviation area [-2; 2] for a fold change ≤3 lies within the solid lines. The gene is considered to be equivalently expressed, if the symmetrical confidence interval is a part of the deviation area and contains 0 in it. The variation in expression of the 13 reference genes was compared between benign vs. borderline and malignant tumours **(A)**, benign and borderline vs. malignant tumours **(B)**, benign vs. malignant tumours **(C)**, mucinous vs. serous benign and borderline tumours **(D)**, and serous vs. endometrioid malignant tumours **(E)**.

Further, we applied the Equivalence test including both cut-offs of 2-fold and 3-fold expression change to identify the best candidates according equivalent expression in group-wise comparison (Figure 2) [8]. The Equivalence test criteria at 3-fold expression change were fulfilled for IPO8, RPL4, RPL30, GUSB, TBP, RPLPO, ACTB, ABL1, and CDKN1A in all subgroups (Table 6). GAPDH was stably expressed only in two out of the five subgroups, followed by HPRT1, HSB90AB1, and PPIA that were equivalently expressed in three subgroups using cut-off of 3. However, IPO8 was the only gene with equivalent expression within 2-fold change in all subgroups.

**Table 6:** Expression stability of the candidate RGs analysed by equivalence test

	**BE × BO+MA**	**BE+BO × MA**	**BE × MA**	**Ser × Muc (BE+BO)**	**Ser × End(MA)**	**Total passes 2-fold/3-fold**
ABL1	0 /1	0 /1	0 /1	1 /1	0 /1	1 /5
ACTB*	0 /1	0 /1	0 /1	1 /1	0 /1	1 /5
CDKN1A	0 /1	1 /1	0 /1	0 /1	0 /1	1 /5
GADPH	0 /0	0 /0	0 /0	0 /1	0 /1	0 /2
GUSB	0 /1	0 /1	1 /1	1 /1	0 /1	2 /5
HPRT1	0 /1	0 /0	0 /0	0 /1	0 /1	0 /3
HSP90	0 /1	0 /0	0 /0	0 /1	0 /1	0 /3
IPO8*	1 /1	1 /1	1 /1	1 /1	1 /1	5 /5
PPIA	0 /1	0 /0	0 /0	1 /1	0 /1	1 /3
RPL30	1 /1	0 /1	0 /1	0 /1	1 /1	2 /5
RPL4*	1 /1	0 /1	0 /1	0 /1	1 /1	2 /5
RPLPO*	0 /1	0 /1	0 /1	0 /1	1 /1	1 /5
TBP*	1 /1	0 /1	0 /1	0 /1	1 /1	2 /5

The expression within (1) or outside (0) 2-fold/3-fold expression change cut-off and the total number of meeting the cut-off criteria in the five subgroups.

* Genes best-ranked by GeNorm, NormFinder and BestKeeper.

### Interpretation of target genes expression

In order to show the effect of the unstable RGs on the final expression of target genes, GPER and uPAR mRNAs were related to either IPO8 and RPL4, or GADPH and HPRT1 mRNA. The choice of target genes was based on our previous observations that GPER mRNA expression did not show any variation between benign, borderline, and malignant ovarian tumour samples [13], whereas uPAR mRNA was higher in borderline and malignant than benign ovarian tumour samples [14].

**Figure 3:**
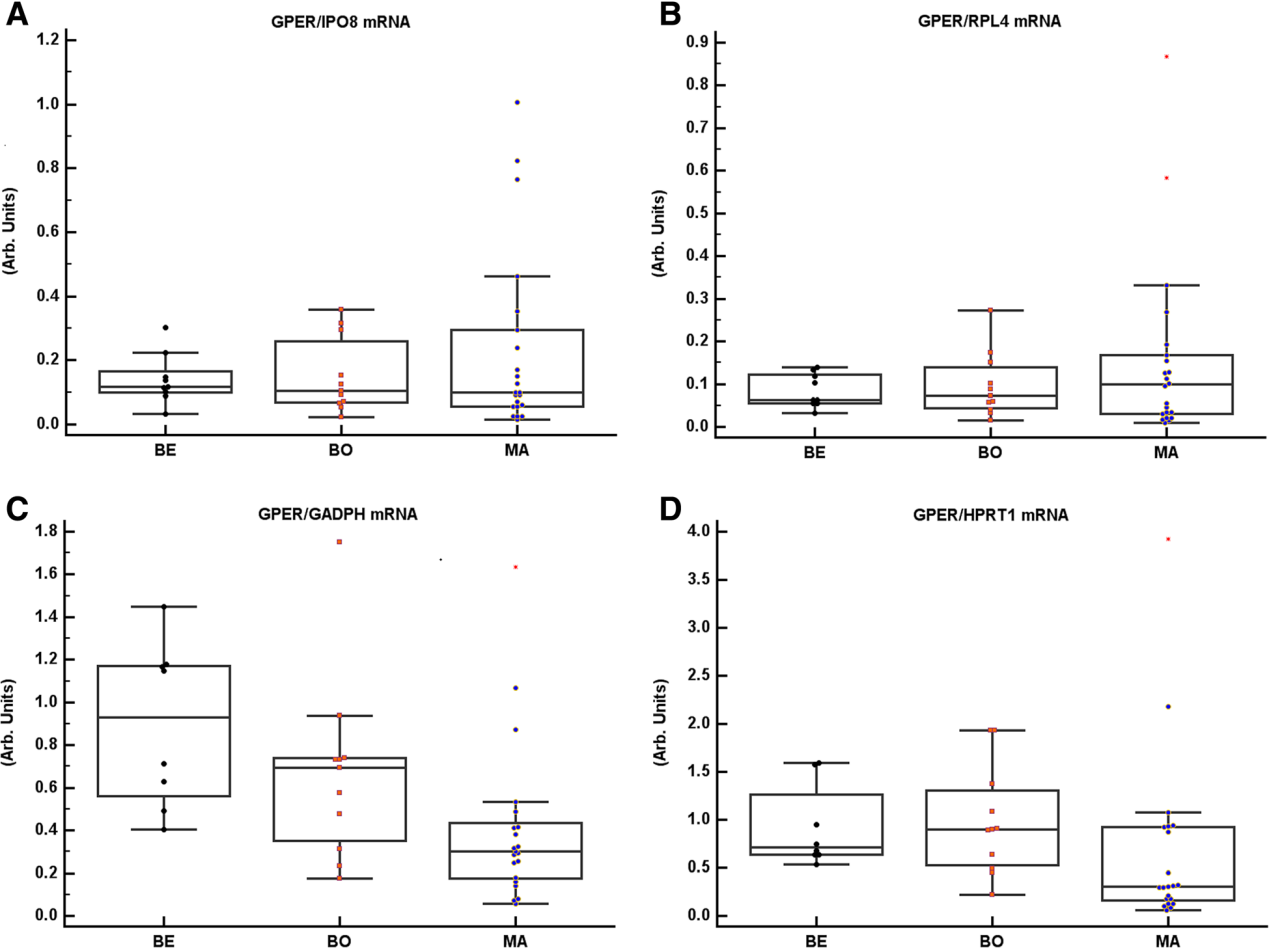
**GPER mRNA assayed and normalized to IPO8, RPL4, GADPH,and HPRT1 mRNA.** Ovarian tumours were sub-grouped according to the histological malignant potential as benign (BE, n = 9), borderline (BO, n = 11) and malignant (MA, n = 22). Normalization to IPO8 and RPL4 showed no significant variation of the GPER mRNA content between BE, BO and MA tumours **(A**, **B)**. In contrast, GPER mRNA was higher in BE/BO compared to MA when normalized to GADPH (p = 0.002) or HPRT1 (p = 0.008) **(C**, **D)**.

**Figure 4:**
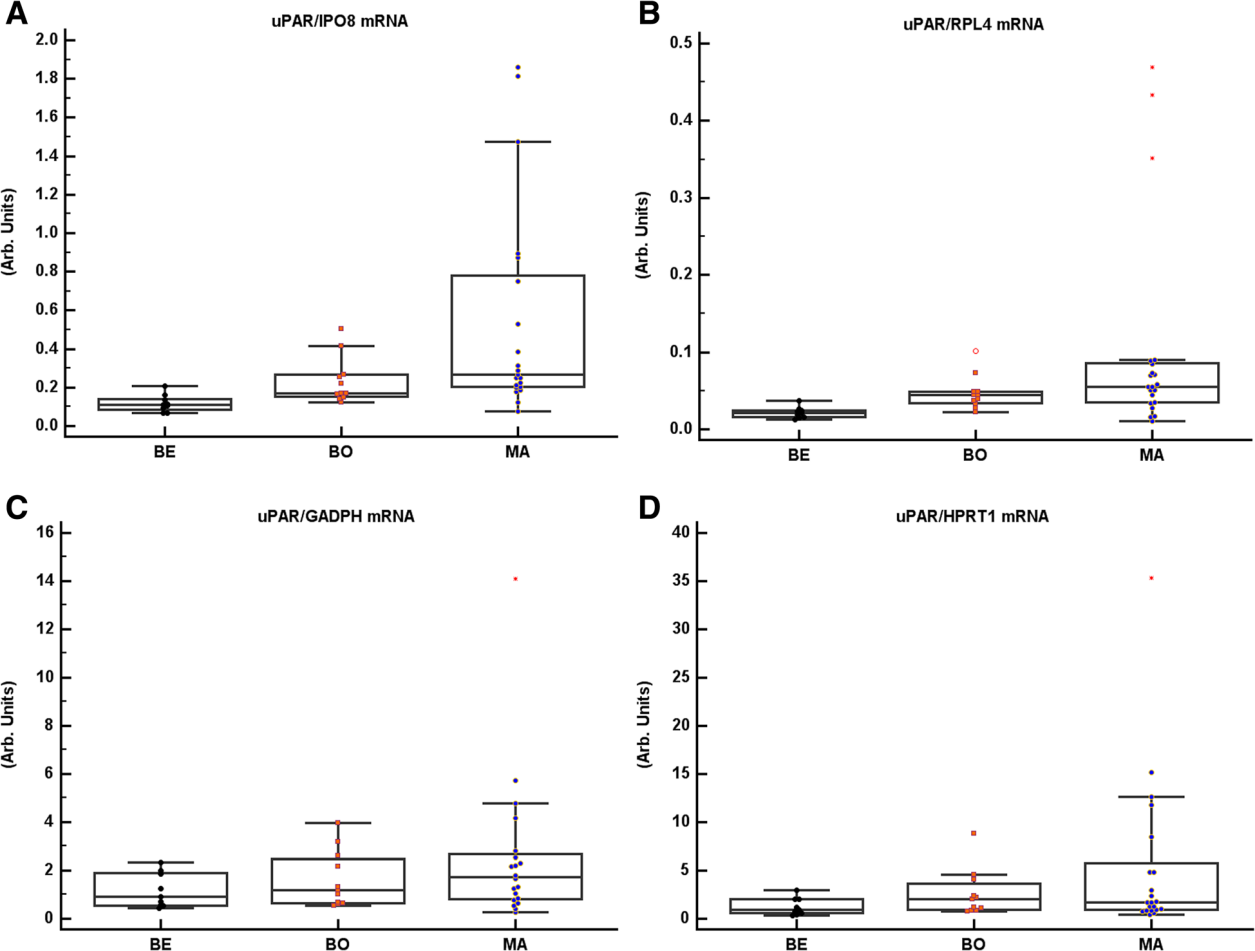
**UPAR mRNA assayed and normalized to IPO8,RPL4,GADPH,and HPRT1 mRNA.** Ovarian tumours were sub-grouped according to the histological malignant potential as benign (BE, n = 9), borderline (BO, n = 11) and malignant (MA, n = 21). uPAR mRNA content was higher in BO/MA than in BE when related to IPO8 (p = 0.003) and RPL4 (p = 0.001) **(A**, **B)**. No significant differences were found in the amount of uPAR mRNA when it was normalized to GADPH or HPRT1 mRNA **(C**, **D)**.

In accordance with our previously published results, the tissue content of GPER mRNA normalized to IPO8 or RPL4 mRNA showed no significant differences between benign, borderline, and malignant tumour samples. In contrast, GPER mRNA normalized to GADPH or HPRT1 mRNA was higher in benign and borderline tumours than in malignant tumours (Figure 3). uPAR mRNA normalized to IPO8 or RPL4 was significantly up-regulated in borderline and malignant tumours as compared to benign tumours, whereas when it was normalized to GADPH or HPRT1 mRNA there were no differences between the tumour groups (Figure 4).

## Discussion

Although RT-qPCR is the most commonly used method for assessing gene expression, in-depth studies of potential reference genes and their expression pattern in ovarian tumour tissue are insufficient. The aim of this study was to identify the most stably expressed RGs, which can be recommended for normalization of RT-qPCR results in benign, borderline and malignant ovarian tumour samples.

We analysed the traditionally used RGs, those reported as being suitable for ovarian tissue, and the four most promising genes from a commercial RG array. Altogether 13 potential reference genes were tested for stability across groups of benign, borderline, and malignant primary ovarian tumours of different histological subtypes. Of the genes studied, IPO8, RPL4, TBP, RPLPO, and ACTB were found to be the most stable according to the statistical applets GeNorm, NormFinder and BestKeeper. Our findings on RPL4, RPLPO, and TBP in a Scandinavian population are in accordance with previous reports in Asian populations [4,6]. In contrast, our results did not support PPIA as suitable RG, which has been observed previously [4]. With regard to the heterogeneity of ovarian tumour materials and different ranking results produced by the commonly used statistical approaches, we decided to further employ the Equivalence test in our analysis. By applying strict criteria in the Equivalence test, i.e. only allowing a 2-fold change of expression, we could identify IPO8 expression as the most stable of all candidate genes tested.

We included IPO8 in our study because it showed low variation in expression between the benign and the malignant sample in the commercial array. This gene was equivalently expressed across the tumour subgroups of different malignant potential and histology. IPO8 is a Ran-binding protein mediating nuclear import [15] and has been already reported stably expressed in lung tissues [16], gliomas [17], and colon cancer [18].

The second best RG for group-wise comparison, RPL4, encodes a protein that is a component of the 60S ribosome subunit [19]. Apart from ovarian tissue, it has previously been recommended as RG in combination with PGK1 for exfoliated cervical cells [20]. RPLPO, another gene from the ribosomal protein family, had stable expression in HPV-positive as in HPV-negative cervical samples [21] and in tamoxifen or estrogen treated breast cancer cells [22]. TBP, a key regulator of gene expression, has previously been identified as a suitable RG for expression studies on human hepatitis B virus-related hepatocellular carcinoma [23], human renal cell carcinoma [24], and glioblastomas [17]. RPLPO and TBP also belonged to one of the most stably expressed genes in breast carcinomas [25].

Two other candidates that have not previously been tested as RGs in ovarian tumour tissue, ABL1 and CDKN1A, were selected from the commercial gene array. Both genes satisfied the Equivalence test at 3-fold expression change. ABL1, originally identified as a homologue of the transforming gene of the Abelson murine leukemia virus, is a proto-oncogene, which has been implicated in mitogenesis, regulation of gene transcription, and inhibition of apoptosis [26]. Nucleotide polymorphism in the ABL1 gene has been associated with risk of ovarian cancer [27]. CDKN1A (also known as p21) was initially described as an inhibitor of cancer cell proliferation [27]. However, recent studies suggest that it has dual functions since it also may promote tumour progression [28] and be associated with cisplatin resistance in ovarian cancer [29].

According to BestKeeper and Equivalence test criteria, we found that GADPH had the worst expression stability in our set of ovarian tumour samples. Similar unfavourable results were obtained for HPRT1. These observations are in line with previous studies on other tissue types that have discouraged use of GADPH and HPRT1 as RGs for clinical lung specimens [16] and renal cell cancer [24]. Most recently, a microarray study identified a group of genes highly correlated to GADPH up-regulation in various solid tumours, which were and proportionally associated with advanced stages [30]. Previous reports on GADPH in ovarian tissue have either pointed out higher expression in malignant than in benign tumours and normal tissue [6], or not meeting the GeNorm stability criteria [4]. We further demonstrated that employment of GADPH or HPRT1 for normalization resulted in erroneous conclusions on expression of target genes.

To our knowledge, this is the first report on RGs in ovarian tumours that include borderline tumours in addition to benign and malignant tumours. Since they are considered a non-invasive pre-stage of molecular type I ovarian cancer, it is important to include them in any study on biomarker discovery [31].

Ovarian cancer comprises tumours of different morphology and pathogenesis, which may have different gene expression profiles [32]. Therefore we wished to see whether the histology of ovarian tumours influences the stability of RGs. Thus, in contrast to the previous studies conducted exclusively on serous malignant tumours, our study also included mucinous and endometrioid tumours. However, small number of samples in some groups limited the comparisons that could be performed.

## Conclusions

In conclusion, thorough statistical evaluation of our 13 candidate RGs identified IPO8 followed by RPL4 as the most suitable for the normalization of gene expression data in benign, borderline, and malignant ovarian tumours. For the first time, IPO8 is presented as the best normaliser for gene expression studies on ovarian tumour tissue with heterogeneous histology when used as a single RG. Neither GADPH nor HPRT1 should be used as RGs for ovarian tissue studies, because of poor expression stability. Normalizing to these genes may erroneously influence the quantification of the target gene(s) and hence reduce the reliability of the RT-qPCR results.

## Abbreviations

RT-qPCR: Quantitative real-time reverse transcription-polymerase chain reaction; RG: Reference gene; IPO8: Importin 8; RPL4: Ribosomal protein 4; GADPH: Glyceraldehyde-3-phosphate dehydrogenase; HPRT1: Hypoxanthine phosphoribosyl transferase 1.

## Competing interests

The authors declare that they have no competing interests.

## Authors’ contributions

ZK carried out the gene expression experiments and drafted the manuscript. AA performed the statistical analysis. BC drafted the manuscript. SH contributed methodological know-how. EK participated in the study design and drafted the manuscript. All authors read and approved the final manuscript.
